# Community Structure and Functional Annotations of the Skin Microbiome in Healthy and Diseased Catfish, *Heteropneustes fossilis*

**DOI:** 10.3389/fmicb.2022.856014

**Published:** 2022-02-28

**Authors:** Shirin Sultana, Md. Nasir Khan, Muhammad Shahdat Hossain, Jingcheng Dai, Mohammad Shamsur Rahman, Md. Salimullah

**Affiliations:** ^1^Aquatic Animal Health Group, Department of Fisheries, University of Dhaka, Dhaka, Bangladesh; ^2^Fisheries Biotechnology Division, National Institute of Biotechnology, Dhaka, Bangladesh; ^3^School of Life Sciences and Technology, Wuhan Polytechnique University, Wuhan, China; ^4^Molecular Biotechnology Division, National Institute of Biotechnology, Dhaka, Bangladesh

**Keywords:** skin microbiota, metagenomics, aquaculture, catfish, *Heteropneustes fossilis*

## Abstract

The skin mucosa of fish serves as a primary barrier against pathogens. In lesion sites in diseased fish, the mucosal barrier is expected to be compromised, with a substantial presence of potential pathogens. An understanding of the skin microbiome and its functional repertoire would provide important insights into host-microbe interactions, which has important implications for prophylactic measures in aquaculture. This study revealed the skin microbiomes and their functional annotations from healthy and diseased stinging catfish (*Heteropneustes fossilis*) based on 16S rRNA metagenomics. The OTUs consisted of four major phyla, Proteobacteria, Bacteroidota, Actinobacteriota and Firmicutes. Among members of the predominant phyla, Proteobacteria were rich in healthy fishes, but Bacteroidota and Firmicutes were significantly differentiated in healthy and diseased fish. The diversified microbiome was high in the skin of healthy fishes and did not significantly differ from that of the diseased groups. At the genus level, *Pseudomonas* showed the highest abundance in healthy fish but was nearly absent in diseased fish, whereas *Flavobacterium* showed the highest abundance in diseased fish. Linear discriminant analysis identified two phyla (Bacteroidota, Firmicutes) and two genera (*Flavobacterium*, *Allorhizobium*) that were consistently identified in diseased fishes. Functional prediction analysis specified that the genes related to physiological functions such as metabolism, immune and digestive systems and environmental adaptations could be highly expressed in diseased fishes. The present study indicates that the compositions, richness and functions of the bacterial community could influence the health status of cultured stinging catfish. Aquaculture-associated pathogenic bacteria may be identified, and preventive measures can be taken for the surveillance of fish health.

## Introduction

Teleosts are associated with diversified microbial communities in the mucosa of the skin, gill and gut. Healthy fish skin harbors an indigenous microbial community that facilitates the homeostasis of host immunity, removes waste products, and outcompetes the colonization of pathogens (Peatman et al., 2015). The epidermal mucus exerts an innate immune response secreted from goblet cells, which act as a primary barrier for pathogenic microbes. In addition, mucus contains antimicrobial peptides, proteases, lysozymes, and lectins that are known to support innate immunity (Dash et al., 2018). The mucus in fish skin is known to entrap and slough off microbes constantly; therefore, skin prevents the colonization of pathogenic microbes. Although fish skin constantly renews mucus, a healthy resident microbial community is maintained in the mucus layer. However, the community structure of the skin microbiome is shaped by multiple factors, such as environmental conditions, health status and host genetics (Van Cise et al., 2020; Xavier et al., 2020). In aquaculture, fish is cultured at high densities, which increases their susceptibility to imbalance in the indigenous microbial community; therefore, understanding the microbial community dynamics relates to aquaculture production. As microbiome of fish plays significant role in their health and development through symbiotic relationship (Collins et al., 2021) though less abundance of community exists in skin mucus compared to gut (Hu et al., 2021).

Disease resistance in fish is dependent on the balance in the diversity of microbiome within the mucus layer (Jani and Briggs, 2014; Lowrey et al., 2015). The skin microbiome defends against colonization by opportunistic pathogens by competing for space or nutrients (Balcázar et al., 2006), generating antagonistic compounds (Boutin et al., 2012; Lowrey et al., 2015), and/or interacting with the host immune system (Kelly and Salinas, 2017). Disrupting homeostasis in skin microbiomes increases the host’s vulnerability to bacterial infections and disease (Cipriano and Dove, 2009; Littman and Pamer, 2011; Mohammed and Arias, 2015; Lockesh and Kiron, 2016). Studies on the bacterial taxa identified on the skin-mucosal surfaces of fish revealed a shift in the microbiome due to stress, allowing potentially pathogenic bacteria to thrive (Boutin et al., 2013; Sylvain et al., 2016). The healthy fish microbiome contain opportunistic pathogens, which can become infectious when hosts are stressed (Austin and Austin, 2007). The fish’s ability to maintain a healthy balance between commensal and opportunistic bacteria in their skin mucus is a significant aspect in protecting health (Gómez and Balcázar, 2008). The decrease in commensal bacteria expressing those functions in fish skin mucus is expected to allow increased abundance of potentially pathogenic organisms.

Skin lesions in fish are the most pressing concern in aquaculture. Ulceration is estimated to kill 1.1–2.5 percent of farmed fish in Norway (Karlsen et al., 2017). Oomycete hyphae are the dominant causative agent of ulcerative mycosis (UM) lesions, but lesions are actually composed of a complex microbial assemblage including many different opportunistic bacteria as well as protozoa (Noga, 2000). However, some ulcerative lesions caused by only bacteria have been observed in UM epidermis (Noga et al., 1991). Skin ulceration and muscular necrosis are dominated by *A. hydrophila*, the freshwater pathogen would cause infection-related mortality in catfish farming systems (Siao et al., 2021). Infections caused the loss of 3 million pounds of farm-raised catfish in Alabama, United States, in 2017, making *A. hydrophila* the most prevalent pathogen in catfish (Siao et al., 2021). However, within the same species of fish, they found different mucosal microbiomes (Butt and Volkoff, 2019). Gaining a better understanding of the skin-mucus microbiota of farmed fish could help to manage the health and diseases of high densities aquaculture species.

The stinging catfish (*Heteropneustes fossilis*) is distributed in Bangladesh, India, Laos, Myanmar, Nepal, Pakistan, Sri Lanka and Thailand (Talwar and Jhingran, 1991). This species is a valuable food fish due to its nutritional value and palatability (Saha and Guha, 1939; Alok et al., 1993). *H. fossilis* is known to be infected with different types of pathogens, such as bacteria, parasites, and fungi (Sahoo and Mukherjee, 1997; Sahoo et al., 1998; Rashid et al., 2008). Bacterial infection, particularly in stinging catfish, can cause heavy mortalities, resulting in severe economic loss. For instance, *Bacillus cereus* caused enormous mortality (the average cumulative mortality on the farm was 5% per day) of stinging catfish in a catfish farm in India in 2019 (Chandra et al., 2015).

Pathogenicity studies based on traditional methods characterized bacterial pathogens in catfishes such as *H. fossilis* and *Clarias batrachus* (Sarkar and Rashid, 2012; Ahammed et al., 2016). However, traditional methods cannot reveal the whole compositions; as a result, many unidentified causative agents for infection remain unknown. Only 1% of the bacterial community can be identified from culture-dependent methods (Amann et al., 1995), while unculturable taxonomic bacterial groups can be efficiently revealed by next-generation sequencing (NGS) (Ringø et al., 2016). Next-generation sequencing methods (metagenomics) allow the profiling of known and unknown microbiome communities in, on and around aquatic animals (Flegel, 2019). Nonetheless, the advantage of the culture-dependent method is that bacterial strains (whether pathogen or with probiotic potential) can be preserved for further experiments, while specific isolates may not be preserved from the culture-independent method.

In this study, we identified bacterial clusters from mucus of healthy specimens and lesions of diseased *H. fossilis* based on both culture-dependent and 16S rRNA metagenomics methods. Bacterial compositions, richness and diversity were analyzed and compared between healthy and diseased groups. As fish immunity is intimately related to a core bacterial community, we also reported bacterial gene functions related to disease and important physiological functions. To the extent of our knowledge, this is the first report describing and comparing the mucosal bacterial community in diseased and healthy stinging catfish.

## Materials and Methods

### Sample Collection and Preparation

**FIGURE 1:**
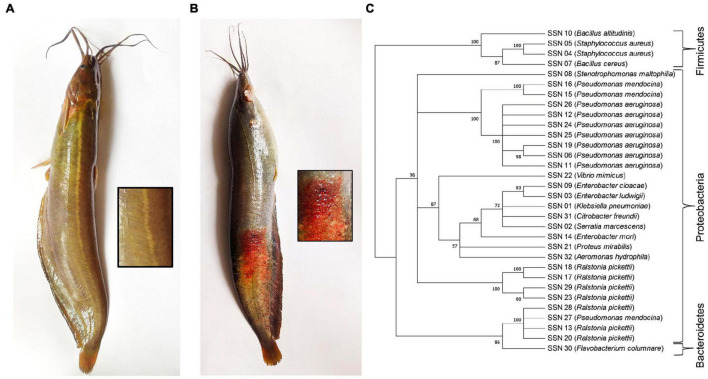
**(A)** Healthy and **(B)** diseased *H. fossilis*. **(C)** The Neighbor-Joining phylogenetic tree of the 32 bacterial isolates based on partial 16S rRNA gene sequences constructed by MEGA X.

Healthy and diseased fishes were collected from the Mymensingh (M), Narsingdi (N), and Dinajpur (D) districts of Bangladesh from October 2019 to January 2020. The samples were collected from selected farm ponds during immediate outbreaks of disease with skin lesions observed on a few fishes. A total of 54 fishes, including 27 healthy (Figure 1A) and 27 diseased (Figure 1B), were collected from the three sources. After aseptic collection of the fishes, each fish was kept in a sterilized zipper bag with proper labeling and brought into the laboratory in an ice box for further analysis. Sample collection details are presented in Table 1. The diseased fish from all locations showed clinical signs of lesions (including ulcers) on the body surface and around the mouth and tail and fin rot. These clinical signs were also reported in stinging catfish (Rashid et al., 2008), Indian catfish (Thomas et al., 2013; Sarjito et al., 2018), and Atlantic salmon (Karlsen et al., 2017). The sampled fish without clinical signs of ulcers were considered to be in good health.

**TABLE 1:** Sample information with collection time and sources.

Sample collection date	Sources	Samples	Replications	Average length (cm)	Average weight (gm)
October, 2019 to January, 2020	Dinajpur (D)	Healthy	ND11, ND12, ND13, ND21, ND22, ND23, ND31, ND32, ND33	15.52	13.88
		Diseased	DD11, DD12, DD13, DD21, DD22, DD23, DD31, DD32, DD33	14.30	13.12
	Narshingdi (N)	Healthy	NN11, NN12, NN13, NN21, NN22, NN23, NN31, NN32, NN33	14.81	12.62
		Diseased	DN11, DN12, DN13, DN21, DN22, DN23, DN31, DN32, DN33	13.59	13.08
	Mymensingh (M)	Healthy	NM11, NM12, NM13, NM21, NM22, NM23, NM31, NM32, NM33	16.15	13.91
		Diseased	DM11, DM12, DM13, DM21, DM22, DM23, DM31, DM32, DM33	14.92	13.39

### Culture-Dependent 16S rRNA Gene Sequencing

Epidermal skin mucus from healthy and wounds from diseased stinging catfish were scraped with separate aseptic scalpels to collect the debris/mucus surface of the wound. The samples were enriched (to increase a small number of desired organisms to detectable levels) in alkaline peptone water (APW) in separate sterile conical flasks and incubated overnight in a shaking incubator (Thermostable IS-20R, South Korea) at 37°C. The enriched solutions were serially diluted with physiological saline (0.9% NaCl). Isolation of bacteria was performed by inoculation and culture on nutrient agar (Oxoid, England). Colony pigment, size, shape, elevation, opacity and edges were observed carefully and ensured the purity of the isolates by culturing sequentially in the same medium for glycerol stock generation and molecular identification.

DNA was extracted from the isolated bacteria by the phenol chloroform method. After DNA extraction, the concentration and purity were measured in a Nanodrop spectrophotometer (Thermo Scientific, United States) at 260/280 ratio. A set of universal primers to detect 16S rRNA (27F 5′-AGA GTT TGA TCC TGG CTC AG-3′ and 1492R 5′-AAG GAG GTG ATC CAG CC-3′) (Weisburg et al., 1991) were used for amplification. Polymerase chain reaction (PCR) was performed in a 25-μl total volume containing 12.5 μl of 2x premix (Maximo), 1 μl of forward primer/10 pmole, 1 μl of reverse primer/10 pmole, 8.5 μl of nuclease-free water and 2 μl (50 ng/μl) of DNA template.

The PCR thermal profile was set as the initial denaturation of 1 cycle at 94°C for 5 min followed by 30 cycles of denaturation at 95°C for 30 s, annealing at 57°C for 1 min, extension at 72°C for 2 min and final extension at 72°C for 10 min. Amplified products were checked in a 1.5% agarose gel and documented in a gel documentation system. All PCR products were purified using a DNA purification kit (Thermo Scientific, United States). Sequencing of the purified PCR products was performed using a Genetic Analyser 3500. A total of 32 nucleotide sequences were obtained and visualized using BioEdit Software (version 7.0.9.0). Sequences were compared with the available sequences in the GenBank database^1^ and confirmed by BLAST up to 97% similarity. The confirmed sequences were submitted to NCBI GenBank under accession numbers MW857176-77,87,98, MW857245-59, 61-63, 71-73,77,79,81,89, 91, MW916538, and MW926915.

A maximum likelihood (ML) phylogenetic tree was constructed using MEGA software version X (Kumar et al., 2018). The evolutionary distances were computed using the p-distance method (Nei and Kumar, 2000). The evolutionary history of the taxa was inferred using the neighbor-joining method (Saitou and Nei, 1987). The percentage of replicate trees was shown in which the associated taxa clustered with the sum of branch length (Felsenstein, 1985). The evenness of the tree was confirmed by bootstrapping (*n* = 1,000) with the MEGA program (Felsenstein, 1985).

### Culture-Independent 16S rRNA Metagenomics

For the culture-independent method, DNA was extracted by following a previously described protocol (Han et al., 2018) with some modifications. Around 30 μl of skin mucus from healthy (slime) and diseased (lesion) fishes were collected by sterile swab sticks in a 2 ml Eppendorf tube containing 500 μl of distilled water. Total DNA was extracted with the phenol chloroform method and then suspended in 100 μl of TE buffer and stored at −20°C after heating at 50°C for 10 min. There were three replicates for each sample in the healthy groups (Narsingdi-NN, Mymensingh-NM, Dinajpur-ND) and the diseased groups (Narsingdi-DN, Mymensingh- DM, Dinajpur-DD).

All DNA samples were sent for next-generation sequencing to Novogene Bioinformatics Technology Co., Ltd. (Beijing, China) on an Illumina Nova sequencing platform. A specific primer with a barcode (515F-806R) was used to amplify the 16S rRNA gene (V4 region). Fifty-four sequencing files were submitted to NCBI under accession numbers SRX10652409 to SRX10652462. The individual samples were assigned as paired-end sequencing based on unique barcodes and were truncated by cutting off the barcode and primer sequence. The obtained quality data were filtered according to Cutadapt (Martin, 2011). The UCHIME algorithm was applied to eliminate the chimera sequences (Edgar et al., 2011). Sequence analysis was performed using Uparse software, where ≥ 97% similarity was assigned to the same operational taxonomic unit (OTU) (Edgar, 2013). OTU-specific sequences were screened for further annotation by the Silva Database (Quast et al., 2012). The phylogenetic relationship of the dominant bacterial species in healthy and diseased fish groups was aligned using MUSCLE software (Edgar, 2004). The α and β diversity indices were estimated based on Quantitative Insights Into Microbial Ecology (QIIME) (Caporaso et al., 2010). Linear discriminant analysis (LDA) effect size (LEfSe) and biomarkers were identified to investigate different bacterial taxa to the genus level, as described by Segata et al. (2011). PICRUSt (phylogenetic investigation of communities by reconstruction of unobserved states) software was used to align the sequences to infer the genes present in the samples and could be expressed (Langille et al., 2013). Mapping and functional annotations of the genes were performed at different KEGG levels (Kanehisa et al., 2016). Bacterial function differences between healthy and diseased fishes were calculated using Welch’s t test.

## Results

### Metagenomics Incorporates All Culture-Dependent Bacterial Isolates

A total of 143 bacterial isolates were retrieved from healthy skin mucus and diseased lesion cultures. Total isolates were merged into 32 based on similar morphological characteristics of the colonies and confirmed by 16S rRNA sequencing to the genus level. The Proteobacteria phylum was represented at 93.75%, and Firmicutes was represented at 6.25%. Fourteen (14) OTUs were annotated at the genus level for lesions, and 26 OTUs for skin mucus and 8 OTUs were common in lesions and mucus (Supplementary Figure 1). The major identified species were *Pseudomonas aeruginosa* and *Ralstonia pickettii*. A neighbor-joining phylogenetic tree was constructed using the 32 isolated bacterial isolates (Figure 1C). Bacterial genera and phyla obtained from the culture-dependent method were completely recovered by 16S rRNA metagenomics. Metagenomics represented the whole microbial community from the skin mucus of healthy and diseased stinging catfish lesions. Therefore, we proceeded with the metagenomics data to analyze the microbiome composition, diversity and richness, identified the top-scoring genera and predicted their gene functions associated with the skin mucus of healthy fish and lesions of diseased fish.

### 16S rRNA Metagenomics Reveals the Abundance of the Microbiome in Skin Mucus

Sequencing was conducted based on the Illumina Nova sequencing platform, constructing a PCR-free library, and using paired-end sequencing. Taxonomic analysis of the 16S rRNA gene for the V4 region generated an average of 91,754 reads from 27 diseased and 27 healthy fish samples from three sources. After quality control, total sequences clustered into 2,514 OTUs (operational taxonomic units) with 97% identity matched with the Silva132 database, of which a total of 2,174 OTUs were annotated to the database. The percentage of annotations to the boundary level was 86.48%. A total of 1,066 (42.40%) OTUs were annotated to the genus level. The proportions at the phylum, class and order levels were 65.79, 65.08, and 61.93%, respectively. Annotation ratios of 54.85, 42.40, and 12.05% were found at the family, genus and species levels, respectively. Proteobacteria, Bacteroidota, and Firmicutes were found to be the three dominant phyla, and *Psychrobacter*, *Pseudomonas*, and *Flavobacterium* were found to be dominant genera. The dominant species were *Pseudomonas psychrophila*, *Psychrobacter alimentarius*, and *Flavobacterium columnare*. The Venn diagram showed that the diseased group formed 1,573 OTUs, and 2,108 OTUs were identified in healthy groups (Supplementary Figure 2). A total of 1,174 OTUs were shared by both the healthy and diseased groups. Healthy groups formed more OTUs than the diseased groups. The larger amount of exclusive OTUs were found in healthy fishes of Dinajpur (ND) (Supplementary Figure 3). A rarefaction curve was made that reached more than 40,000 reads, indicating the sufficiency of the sequence coverage, while the species rank abundance curve also supported this good coverage by showing a higher relative abundance of the species (Supplementary Figure 4).

### Healthy Stinging Catfish Harbor Richer and More Diversified Microbiomes

**FIGURE 2:**
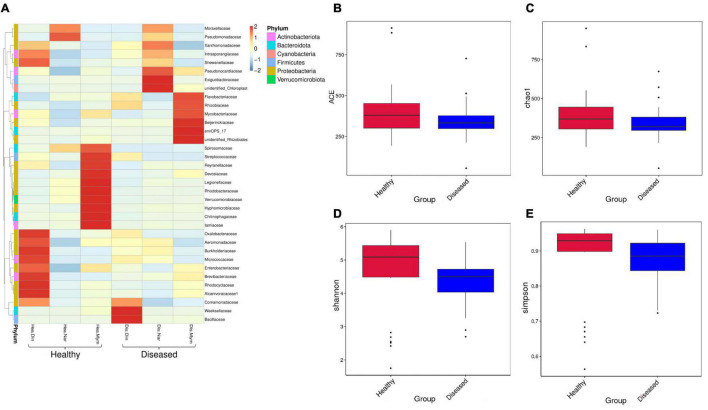
Relative abundance, richness, and diversity of microbiome community in healthy and diseased *H. fossilis*. **(A)** Heatmap of OTU at family level with relative abundance. Columns represent groups of samples, rows indicate family OTUs. The color with key scale represents intensity of OTUs from lower (blue) to higher (red). **(B–E)** Variation of alpha diversity is shown by Boxplots. Non-significant higher microbial diversity in healthy fishes represented in ACE **(B)**, Chao1 **(C)**, Shannon **(D)** and Simpson indices **(E)**.

All the reads yielded nine classifiable phyla, viz., Proteobacteria, Bacteroidota, Firmicutes, Actinobacteriota, Verrucomicrobiota, Cyanobacteria, Fusobacteria, Planctomycetes and Campilobacterota. The most dominant phyla in the healthy (ND, NN, NM) and diseased groups (DD, DN, DM) were Proteobacteria (58.96 and 35.14%), Bacteroidota (29.89 and 17.82%), Firmicutes (4.32 and 2.57%), and Actinobacteria (6.83 and 4.07%). The relative abundance of the most dominant family was higher in healthy fishes (Figure 2A). The associated bacterial classes in the Narshingdi (NN, DN) samples were more diverse than those in the Mymensingh (NM, DM) and Dinajpur (ND, DD) samples.

Additional analyses of α and β diversities between healthy and diseased groups were performed. Non-significant alpha diversity indices indicated that the higher diversity was in healthy groups compared to the diseased groups. We found differences in observed species between the healthy and diseased groups, and other indicators of richness were also found to be higher in the healthy groups by ACE (Figure 2B) and Chao 1 (Figure 2C) analysis. The diversity estimators, Shannon (Figure 2D) and Simpson index (Figure 2E), were also higher in the healthy groups. A rarefaction curve based on OTUs was also constructed to estimate the bacterial diversity. The healthy groups were found to contain a higher level of species richness than the diseased groups. The relative abundance of species diversity was also higher in the healthy groups. Location-wise, the healthy fishes of Dinajpur (ND) showed non-significantly higher diversity and richness than Mymensingh (NM) and were significantly different from Narsingdi healthy (NN) fishes, while the highest diversity and richness were not significant among the diseased fishes, as revealed by the different alpha diversity parameters. The Shannon index was significantly different between disease Narsingdi-healthy Dinajpur (DN-ND), disease Dinajpur-healthy Dinajpur (DD-ND) and healthy Dinajpur-healthy Narsingdi (ND-NN) (*p* < 0.05). There was no significant difference between the groups of infected fishes (*p* > 0.05).

**FIGURE 3:**
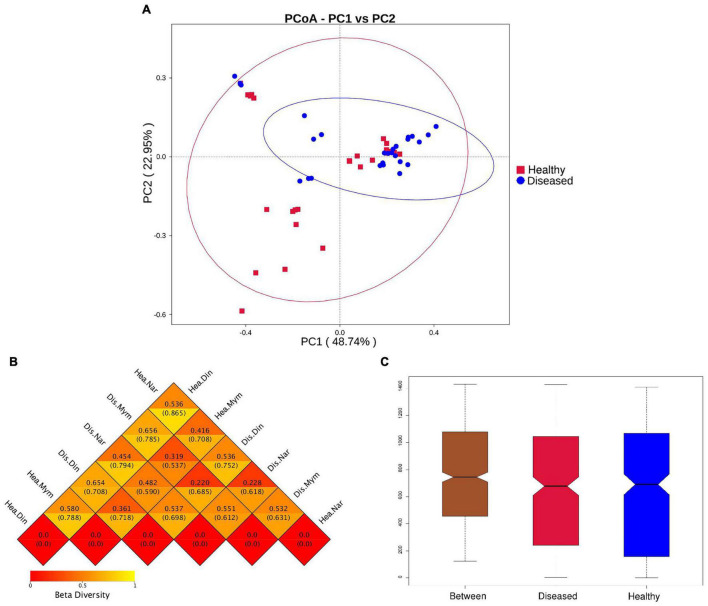
Beta diversity analysis of healthy and diseased group of *H. fossilis*. **(A)** Principal coordinates analysis (PCoA) of bacterial community based on Weighted UniFrac distance matrix. Each blue color dot represents infected fish and each red color dots indicate each healthy fish. **(B)** The heatmap of beta diversity index shows the distance values with lower distance (red) to higher (orange). The upper values represent the Weighted Unifrac distances, and the lower values represent the Unweighted Unifrac distances. **(C)** Intergroup and intragroup analysis of similarity (Anosim). R- value indicates dissimilarities between inter and intra groups and *P* value <0.003 shows that result was statistically significant.

The beta diversity was calculated using weighted and unweighted UniFrac analysis. A three-dimensional scatter plot generated using principal coordinate analysis (PCoA) showed overlapping and scattering of microbial communities (Figure 3A) which is also supported by Non-metric multidimensional scaling plots (NMDS) between the healthy and diseased groups (Supplementary Figure 5). At the species level, the PCoA revealed that PC1 explained 48.74% and PC2 explained 22.95% of the total variance observed in the dataset. Weighted Unifrac and unweighted UniFrac distance index value indicates the difference coefficient between the healthy and diseased communities. In our study, the highest distance value (0.865) was found in healthy fishes of the Narsingdi (NN) and Dinajpur (ND) samples (Figure 3B), followed by diseased Narsingdi (DN) and healthy Dinajpur (ND) (0.794) and healthy Mymensingh and Dinajpur (NM and ND) (0.788). One-way analysis of similarities (ANOSIM) between samples collected from different locations showed differences between groups (*R* = 0.141, *p* = 0.003) (Figure 3C). Values of R_*ANOSIM*_ between 0.25 and 0.75 indicate some degree of overlap while values < 0.25 indicate two group of communities are hardly distinguishable (Flynn et al., 2017). In our study, pairwise dissimilarities were found to be significant within the groups except for healthy and diseased fishes collected from Narsingdi (*R* = 0.1135, *p* = 0.109) (Supplementary Table 1).

### Diseased and Healthy Fishes Maintain Different Microbiome Profiles

**FIGURE 4:**
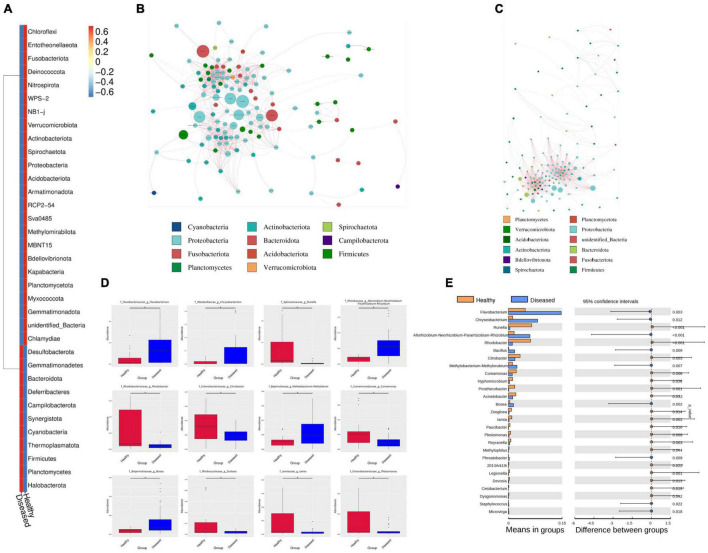
The difference between healthy and diseased of *H. fossilis* at phylum and genus level by network comparison and *t*-test. **(A)** The heatmap shows phyla abundance between groups. The key color scale showed the intensity range blue to red (lower to higher) of each phylum. Network comparison at genus level between diseased **(B)** and healthy **(C)**. Each node represents each genus and node size indicates the abundance of the respective group. **(D)** The Boxplot showed the abundance and depletion of genera between groups. **(E)** The *P* values < 0.05 define a statistically significant variation at phylum and genus level.

Healthy groups formed more OTUs than the diseased groups. Phylum-level analysis revealed differences in abundance between the healthy and diseased groups. The phyla Proteobacteria, Bacteroidota and Actinobacteriota were dominant in healthy fishes, whereas the diseased groups dominated by Proteobacteria, Bacteroidota and Firmicutes (Figure 4A).

Significant differences were detected in the Bacteroidota and Firmicutes phyla between the healthy and diseased groups. Both phyla were significantly more abundant in the healthy and diseased groups (*p* < 0.005). In the diseased groups, the dominant Firmicutes contributed major microbiota in Narsingdi (DN) sources, which were also found in diseased fishes of Dinajpur (DD) and a small number of microbes in Mymensingh (DM). This phylum was rarely present in healthy fishes (NN, ND, NM). The Proteobacteria phylum was insignificantly more abundant in the healthy group than in the diseased group. This phylum was abundant in both the healthy and diseased groups of Narsingdi (NN and DN). Similarly, Actinobacteria dominated in healthy fishes of Dinajpur (ND) and diseased fishes of Narsingdi (DN). An unidentified phylum was rich in healthy fishes of Mymensingh (NM), which significantly differed from all other sources.

Our study showed that many families and genera were common in fishes in the healthy and diseased group fishes, but their abundance varied greatly between these groups. This common pattern at the order and class levels also exist among the host species. The Flavobacteriaceae, Moraxellaceae, Pseudomonadaceae, Exiguobacteriaceae, and Weekellaceae families were abundantly present in different sources of diseased fishes. Flavobacteriaceae varied slightly between infected fishes of Dinajpur (DD) and Mymensingh (DM) but was rarely present in Narsingdi (DN). Moraxellaceae and Pseudomonadaceae were highly abundant in the diseased fishes of Narsingdi (DN). The families Rhodobacteraceae, Reyranellaceae, and Spirosomaceae were highly abundant in healthy fishes.

**FIGURE 5:**
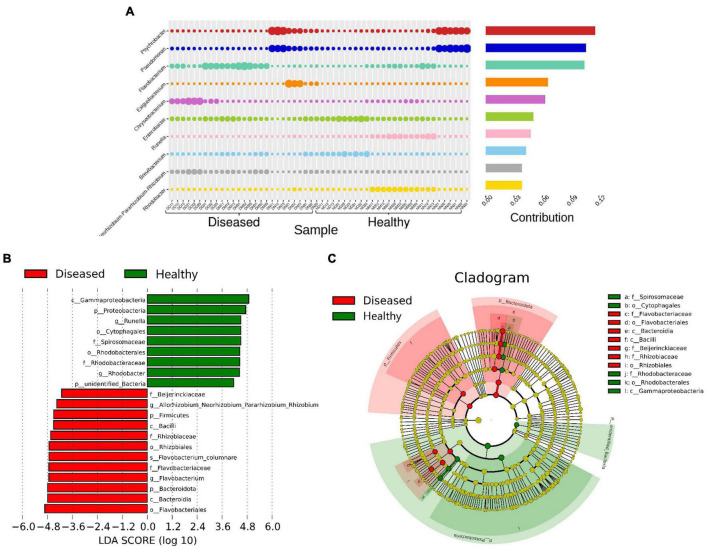
Most abundant bacterial communities that differentiate healthy and diseased *H. fossilis*. **(A)** The percentage similarity analysis (SIMPER) shows intensity of bacteria at the genus level. **(B)** Histogram based on Linear discriminant analysis (LDA) scores represents distinguishing bacterial communities. **(C)** Circular Cladogram showed taxonomic distribution of bacterial abundance between two groups. Increased abundance of OTUs in healthy group contributed by Proteobacteria, Runella and Rhodobacter, while diseased fishes had increased abundance of Flavobacteriales.

Network comparison and putative genera identification were performed between the diseased and healthy groups at the genus level, which showed that the healthy groups had more network diameters than the diseased groups (12 *vs.* 8) (Figures 4B,C). A higher clustering coefficient (CC), average path length (APL), and average degree (AD) in healthy groups specifying that more significant correlations existed between the genera in healthy fishes. The members of the genera *Psychrobacter*, *Pseudomonas*, *Flavobacterium*, *Exiguobacterium*, *Chryseobacterium*, *Enterobacter*, and *Methylobacterium* contributed significantly to the diversity and differentiated the healthy and diseased groups (Figure 4D). *Flavobacterium*, *Methylobacterium*, and *Chryseobacterium* were significantly high in the infected groups (*p* < 0.005) (Figure 4E). In addition, different pathogenic genera, such as *Citrobacter*, *Aeromonas*, *Ralstonia*, and *Rickettsia*, were also present in diseased fishes. Conversely, communities of the *Psychrobacter*, *Pseudomonas* and *Enterobacter* genera were abundant in healthy fishes from the three sources (Figure 5A). *Psychrobacter* was highly abundant in Narsingdi (NN), absent in Dinajpur (ND) and rarely present in Mymensingh (NM). Similarly, *Pseudomonas* was rich in Narsingdi (NN) and rarely present in healthy fishes of Dinajpur (ND) and Mymensingh (NM). The top 10 genera that mostly contributed to the healthy and diseased groups are shown in Table 2.

**TABLE 2:** Percentages of top 10 genera in healthy and diseased stinging catfishes.

Genus	Healthy	Diseased
*Allorhizobium*	4.78%	3.37%
*Brevibacterium*	6.49%	0.39%
*Chryseobacterium*	1.19%	8.14%
*Enterobacter*	9.42%	7.38%
*Exiguobacterium*	0.23%	4.92%
*Flavobacterium*	4.68%	14.74%
*Neorhizobium*	1.60%	6.01%
*Pararhizobium*	6.18%	1.09%
*Pseudomonas*	11.69%	7.35%
*Psychrobacter*	10.43%	9.12%

Linear discriminant analysis (LDA) based on the LEfSe algorithm was computed to categorize taxonomic differences between the healthy and diseased fish (Figure 5B). A logarithmic LDA score (log 10) cut-off of 4.8 revealed that healthy fish sheltered Proteobacteria, *Runella* and Rhodobacter, while increased abundances of Firmicutes, Flavobacteriales, and *Flavobacterium columnare* were observed in the diseased groups (Figure 5C).

### Pathogens From Mucus and Lesions Influence Important Physiological Functions

**FIGURE 6:**
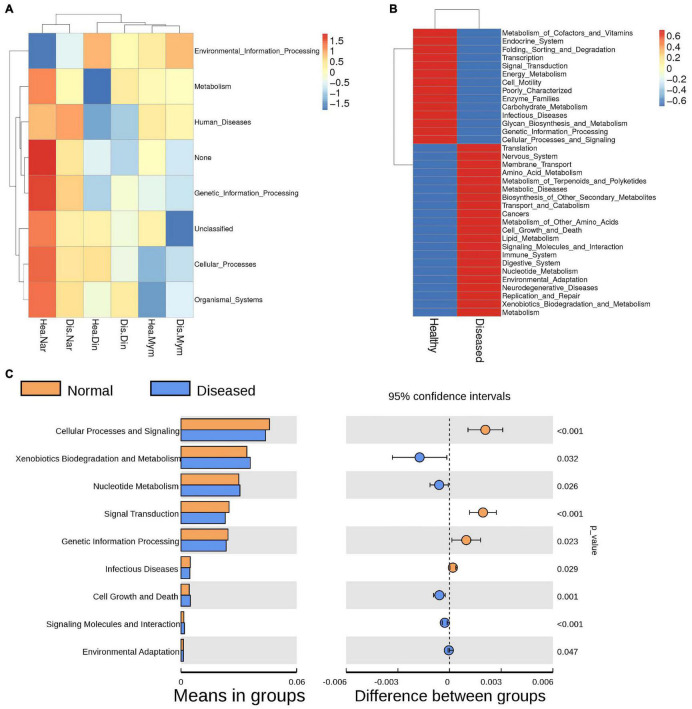
Functional annotations predicted by KEGG pathway analysis of the microbiomes in healthy and infected fishes. **(A)** Heatmap shows the changes in KEGG level 1 due to microbial functions. **(B)** Comparisons of genes responsible for physiological functions between the groups analyzed by KEGG level 2. **(C)** The Welch’s t-test of the functional predictions generated by the KEGG level 2. *P* values (< 0.005) represents the statistically significant variation between the groups.

Functional changes were obvious due to the changes in microflora between healthy and infected fishes. The significance of these changes for disease creation and potential benefits was predicted by PICRUSt. The level 1 KEGG pathway revealed that predicted functions related to metabolic pathways and environmental and genetic information processing were not significantly different between the groups (Figure 6A). The relative abundance of metabolic pathways accounted for a total of 47.88%, where 23.90% of genes accounted for healthy fish metabolism and 23.98% of genes contributed to the metabolic activities of diseased fishes. The KEGG level two pathway indicated that genes for membrane transport and amino acid and carbohydrate metabolism were predicted to be slightly higher in the diseased groups, with average values of 13.96%, 10.23%, and 9.83%, respectively. Functional prediction analysis indicated that genes related to the immune system, digestive system, environmental adaptation, neurodegenerative disease, and xenobiotic biodegradation could be higher expressed in diseased fishes (Figure 6B). KEGG level 3 functional pathway analysis of genes related to different amino acids and enzymes were predicted to be higher in infected fishes than in healthy fishes (Supplementary Figure 6). The pathways connected with infectious diseases, metabolic functions, and processing of genetic information were significantly higher in the healthy group (*p* < 0.05) (Figure 6C).

## Discussion

The outbreak of any disease is strongly related to the interactions between fish pathogens and the environment (Noga, 2000). It has been shown that single or mixed bacterial strains are responsible for approximately 60% of infectious diseases in fishes (Tucker and Hargreaves, 2004; Mohammed and Peatman, 2018). In the current study, we identified a mixture of pathogenic bacteria from the lesions of diseased *H. fossilis* fishes by a culture-dependent method, which was later confirmed by culture-independent 16S rRNA sequencing.

The mortality of catfish due to bacterial disease is severe, making them prone to disease outbreaks in winter due to the favorable temperature for disease outbreaks (Mohammed and Peatman, 2018). By analyzing healthy and diseased fishes from three sampling sites, we found 2,174 OTUs annotated for genus. Among them, the healthy fishes had more OTUs than diseased fishes. In general, healthy fishes have a more diverse bacterial community that play different functions, whereas diseased fishes show fewer and abnormal physiological functions. Non-significant difference in OTUs numbers was reported in healthy and diseased yunlong grouper fish (Ma et al., 2019).

The bacterial abundance of Gammaproteobacteria was reported from the skin of European catfish (Chiarello et al., 2019). We observed four major phyla, Proteobacteria, Bacteroidota, Actinobacteriota and Firmicutes, in the mucus of the healthy and diseased groups. These common phyla have also been reported in many fish species, including catfish skin (Wu et al., 2010; Lowrey et al., 2015; Zhang et al., 2017; Ma et al., 2019). The dominant families of these phyla, such as Enterobacteriaceae, Bacillaceae, and Clostridiaceae, were found to be pathogenic and transmissible to humans through water (Mukherjee et al., 2016). Both pathogenic and beneficial members from these phyla were identified in the studied healthy and diseased fishes. In the diseased groups, the increased abundance of the Firmicutes phylum indicates that members of this phylum can cause fatal infection in *H. fossilis* by virtue of the interaction between the pathogenic bacteria. The increase phenomenon of the Firmicutes phylum has also been shown in other species by other researchers (Lowrey et al., 2015; Kim et al., 2017).

Harmonized microbiome composition, including beneficial and opportunistic pathogens from fish skin and the environment, has been reported in previous studies (Georgala, 1958; Horsley, 1977; Ahmed et al., 2018). In a healthy environment, beneficial microbes fight against opportunistic pathogenic organisms’ virulence and prevalence through the secretion of antimicrobial compounds by a process known as antibiosis (Boutin et al., 2012; Lowrey et al., 2015; de Bruijn et al., 2018). Similar to mammals and plants, imbalanced microbial communities cause infection and diseases (Sagvik et al., 2008; Stecher et al., 2013; Turner et al., 2013; Romero et al., 2014; Montalban-Arques et al., 2015; Moya and Ferrer, 2016; de Bruijn et al., 2018) when fishes face adverse environmental conditions under stress. Exposure to prolonged stress inhibit the immune system and accelerate potential pathogenic organisms thereby, shifting the bacterial community (Hu et al., 2021). In the present study, the genera *Flavobacterium*, *Exiguobacterium*, *Chryseobacterium*, *Rhizobium*, *Bacillus* and *Methylorubrum* presented an increased pattern in the diseased groups, whereas the *Runella*, *Rhodobacter*, *Pseudomonas, Citrobacter*, *Comamonas*, *Hyphomicrobium*, and *Prosthecobacter* genera were depleted in the diseased groups and increased in the healthy groups (Supplementary Figure 7). Beneficial *Pseudomonas* species produce vitamin B12 (Fang et al., 2017), which helps the host maintain normal growth (Arai et al., 1972; Ikeda et al., 1988; Ma et al., 2019) and physiological functions. The increased mortality rate of diseased *H. fossilis* may be associated with the depletion of *Pseudomonas* genera. *Pseudomonas* provides a toxic pollutant-free natural environment and generates compounds that are toxic to other species (Novik et al., 2015). Thus, these beneficial bacteria identified from healthy fish mucus could be used as potent probiotics. Beneficial microbes protect aquaculture species by killing pathogens or preventing the expression of pathogenic virulence factors (Kesarcodi-Watson et al., 2008; van Hai and Fotedar, 2010) through inhibition of their quorum sensing (QS) ability (Flegel, 2019).

The highest abundance of identified genera in the diseased groups suggested that the species of the *Flavobacterium* genus may cause skin lesions in diseased fishes (Supplementary Figure 8). This fact is not unexpected, as *Flavobacterium* is well reported as a dominant fish pathogen (Chiarello et al., 2019) throughout the world, causing diseases with considerable losses in wild and farmed fishes (Wahli and Madsen, 2018). *Aeromonas*, *Columnare*, and *Edwardsiella* are the major bacteria causing diseases in catfish (Zhou et al., 2018). *Exiguobacterium* and *Chryseobacterium*, which were detected in the diseased fish in our experiment, were also reported as pathogens for fishes, reptiles, birds and mammals, including humans (Bernardet and Nakagawa, 2006; Loch and Faisal, 2015). Other pathogenic bacteria, such as *Citrobacter, Enterobacter, Aeromonas, Morganella, Ralstonia, Rickettsia, Pseudomonas, and Acinetobacter*, were identified from healthy and diseased fishes, indicating that they may be opportunistic and beneficial depending on the physiological and environmental conditions. Bacteria identified from diseased fishes can induce opportunistic bacteria to proliferate and inhibit the growth of beneficial microbiome groups. To overcome disease conditions, beneficial bacteria can facilitate host immunity through secretion of antibodies and active antimicrobials by the presence of pathogenic microbe peptides (Ángeles Esteban, 2012; Kelly and Salinas, 2017; Zhang et al., 2018).

Bacterial communities in fishes are subject to different geographical locations and seasons (Sullam et al., 2012; Ye et al., 2014; Zarkasi et al., 2014; Ray, 2016; Tarnecki et al., 2017). We identified non-significant differences in clusters by location, which is also not unusual (Smith et al., 2015). The bacterial community from the phylum Proteobacteria was uniformly present in all locations, but other dominant phyla, Bacteroidota, Actinobacteriota and Firmicutes, probably showed increasing and decreasing patterns in different locations. However, different patterns at the genus level were also observed in our studied samples. The genera *Psychrobacter, Pseudomonas, Flavobacterium, Chryseobacterium, Enterobacter, Ralstonia* and *Brevibacterium* were significantly different in each locations and in each part of the healthy and diseased samples. LDA analysis could differentiate the microbiome clusters and their effects on healthy and diseased fishes, and microbiome clusters were also differentiated in healthy and diseased Yunlong Grouper and shrimp (Ma et al., 2019; Hossain et al., 2021). LDA analysis detected 1 phylum, 3 classes, 6 orders, 4 families and 4 genera; among them, in healthy fishes, *Runella* and *Rhodobacter* had the highest scores, while in infected fishes, *Flavobacterium columnare* and Firmicutes showed the highest scores, similar to the findings of Zhang et al. (2018). Furthermore, principal coordinate analysis also differentiated the cluster of microbial communities, although non-significant, from the diseased and healthy groups.

Functional prediction analysis revealed many functional gene richnesses that are related to metabolism, environmental information processing, immune systems, environmental adaptation and neurodegenerative diseases. Carbohydrate metabolism of the host contributed by intestinal microbiota has been reported in many species (Hooper et al., 2002; Turnbaugh et al., 2006; Brulc et al., 2009; Hess et al., 2011; Zhu et al., 2011). In line with other studies, genes related to increased carbohydrate and amino acid metabolism of the microbiota of diseased fishes were found to be high (Ni et al., 2014; Ma et al., 2019). The physiological process of carbohydrate metabolism also increased with the occurrence of disease. Although we observed differences between healthy and diseased groups for carbohydrate metabolism in the present study, the differences were not significant (Ma et al., 2019). The functions of immune system- and environmental adaptation-related genes were high in infected *H. fossilis* fishes, indicating that infected fishes need more energy to adapt to adverse environments (Zhang et al., 2018).

In the present study, we found higher richness and diversity of the mucosal microbiome in healthy *H. fossilis* than in diseased *H. fossilis*. The beneficial microbiome protects fish from pathogenic organisms and plays an important role in physiological functions. Under adverse environmental conditions, pathogenic bacteria become dominant in skin and affect the overall composition of diseased fish. These pathogens influenced the functions of genes related to metabolism, immune responses, and environmental adaptations of fish and suppressed growth and production. This phenomenon could be important in understanding the synergistic and antagonistic interactions between resident microbes and pathogens in aquaculture species. Overall, our study provides baseline information regarding the early detection of causative agents in *H. fossilis* and helps in taking preventive measures against severe mortality in the species due to the presence of pathogenic microbes.

## Data Availability Statement

The datasets presented in this study can be found in online repositories. The names of the repository/repositories and accession number(s) can be found in the article/Supplementary Material.

## Ethics Statement

The animal study was reviewed and approved by ERB No: NIBREC2018-04, National Institute of Biotechnology Ganakbari, Ashulia, Savar, Dhaka-1349, Bangladesh.

## Author Contributions

MS conceived the project idea. MS, MR, SS, and MH designed the experimental approach to be used. SS and MK collected the samples, provided clinical assessments and defined the sample populations, and generated the laboratory data. MH and JD provided guidance on the analysis of the data. SS and MH collated, analyzed, and interpreted the data. MS and MR provided overall project guidance and support, and edited and reviewed the manuscript. SS wrote the manuscript. All authors provided comments on the manuscript prior to submission.

## Conflict of Interest

The authors declare that the research was conducted in the absence of any commercial or financial relationships that could be construed as a potential conflict of interest.

## Publisher’s Note

All claims expressed in this article are solely those of the authors and do not necessarily represent those of their affiliated organizations, or those of the publisher, the editors and the reviewers. Any product that may be evaluated in this article, or claim that may be made by its manufacturer, is not guaranteed or endorsed by the publisher.

## Footnotes

1
https://www.ncbi.nlm.nih.gov/genbank
