# Supplementary material for: Community Structure and Functional Annotations of the Skin Microbiome in Healthy and Diseased Catfish, Heteropneustes fossilis
Source: Front Microbiol. 2022 Feb 28;13:856014. doi: 10.3389/fmicb.2022.856014 (PMC8918984; doi:10.3389/fmicb.2022.856014)
Supplement: Supplementary file 1 [file Data_Sheet_1.docx]

Supplementary Material

# Supplementary Figures and Tables

## Supplementary Figures


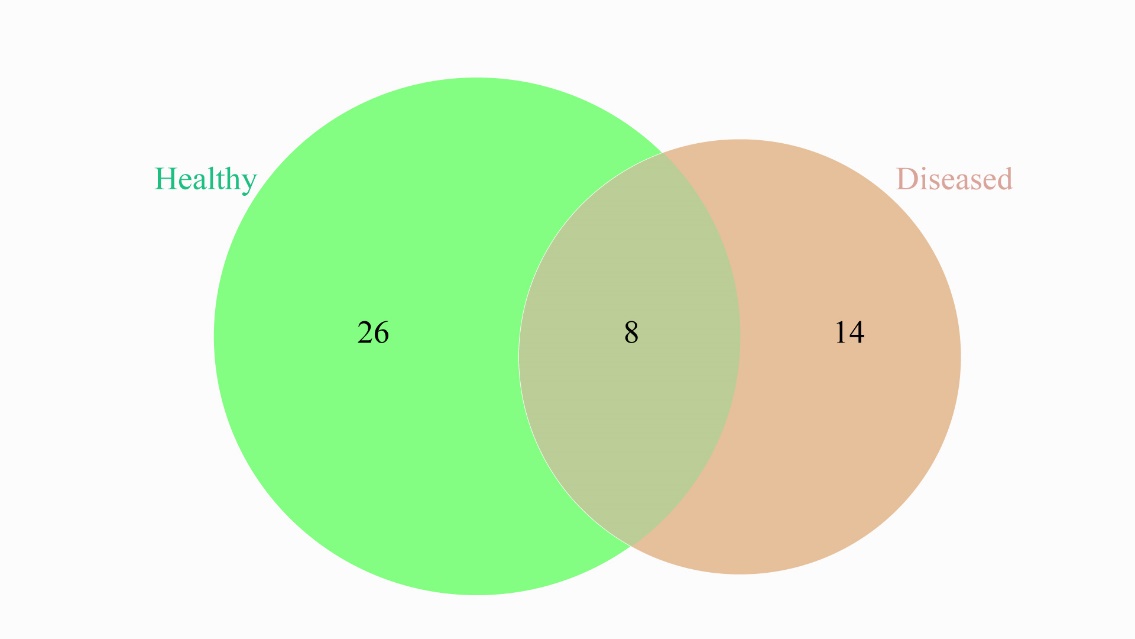


## Supplementary Figure 1. Percentages of bacterial genera identified by culture method from healthy and diseased *H. fossilis*. Healthy group covered 26 OTUs, diseased group covered 14 OTUs and both groups shared 8 OTUs of the total genera.


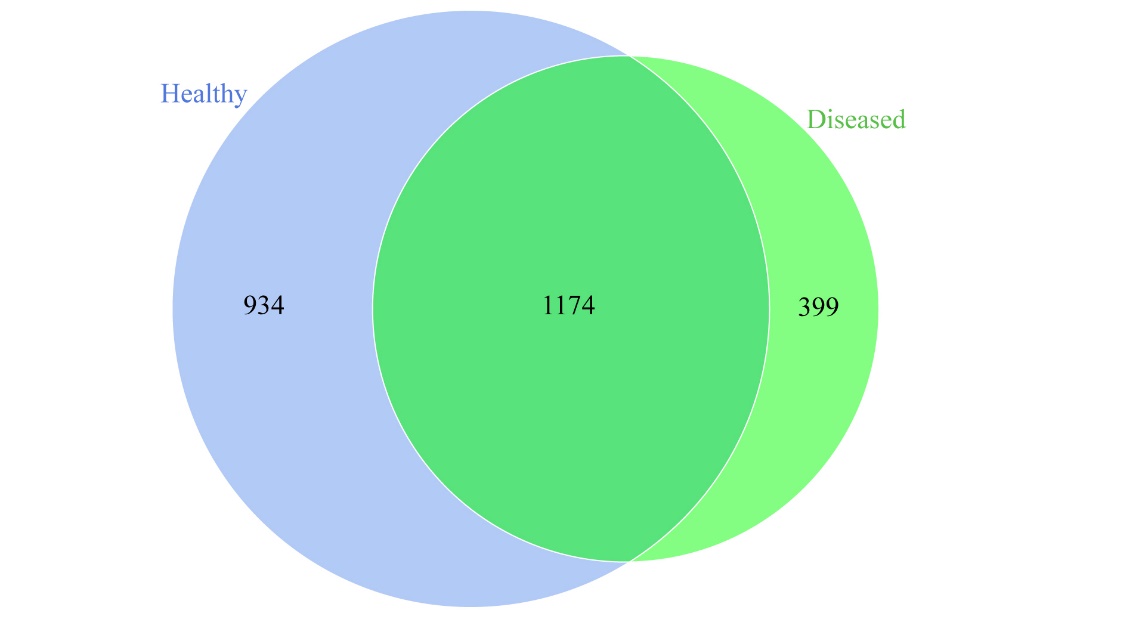


## Supplementary Figure 2. Numbers of OTUs identified by metagenomics analysis from healthy and diseased *H. fossilis*. Healthy group formed 2108 OTUs, diseased group formed 1573 OTUs and both groups shared 1174 OTUs.


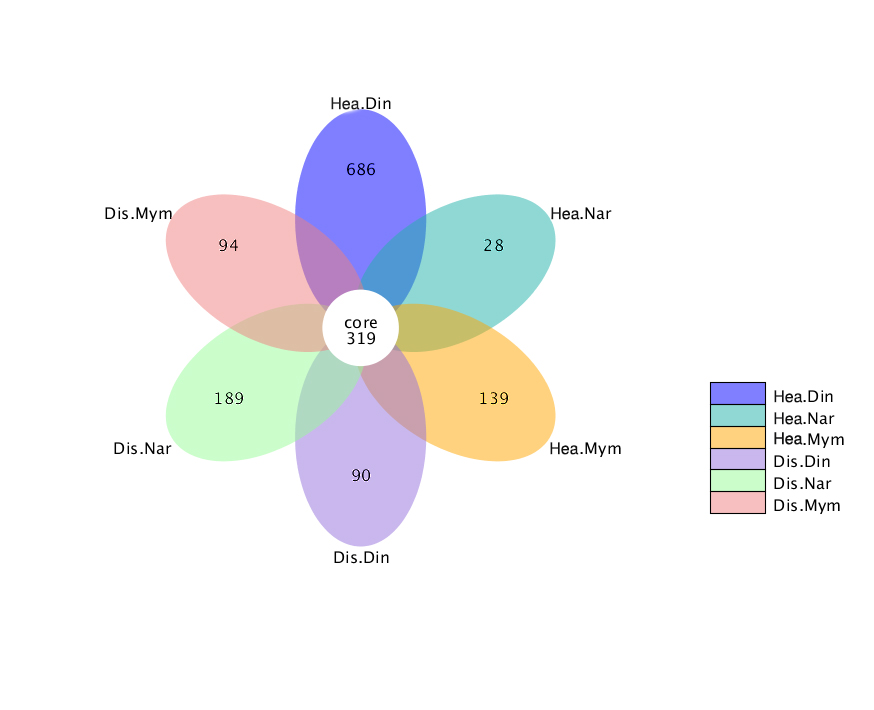


## Supplementary Figure 3. Distribution of OTUs between healthy and diseased groups collected from three sources. The highest OTUs (686) was found in Dinajpur sample of healthy groups, whereas highest (189 OTUs) was found in Narsingdi sample of diseased groups. (Dis: Disease; Hea: Healthy; Din: Dinajpur; Nar: Narsingdi; Mym: Mymensingh)


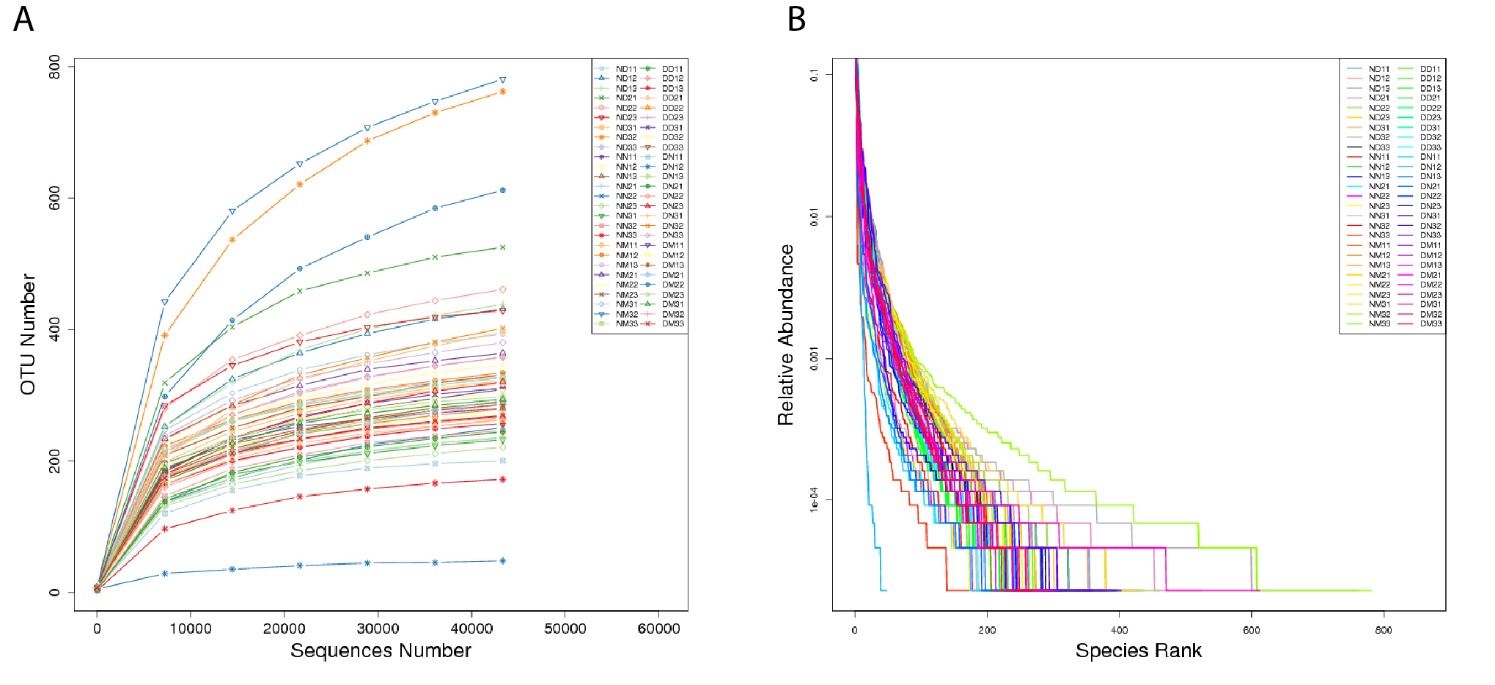


## Supplementary Figure 4. Rarefaction curve and species rank shows quality of the sequence and richness of each sample. (A) Rarefaction curve reached above 40000 reads (B) Species rank abundance curve showed species composition. The more flatter the curve, the more evenness of species composition and the more higher the curve, the more richer of species composition.


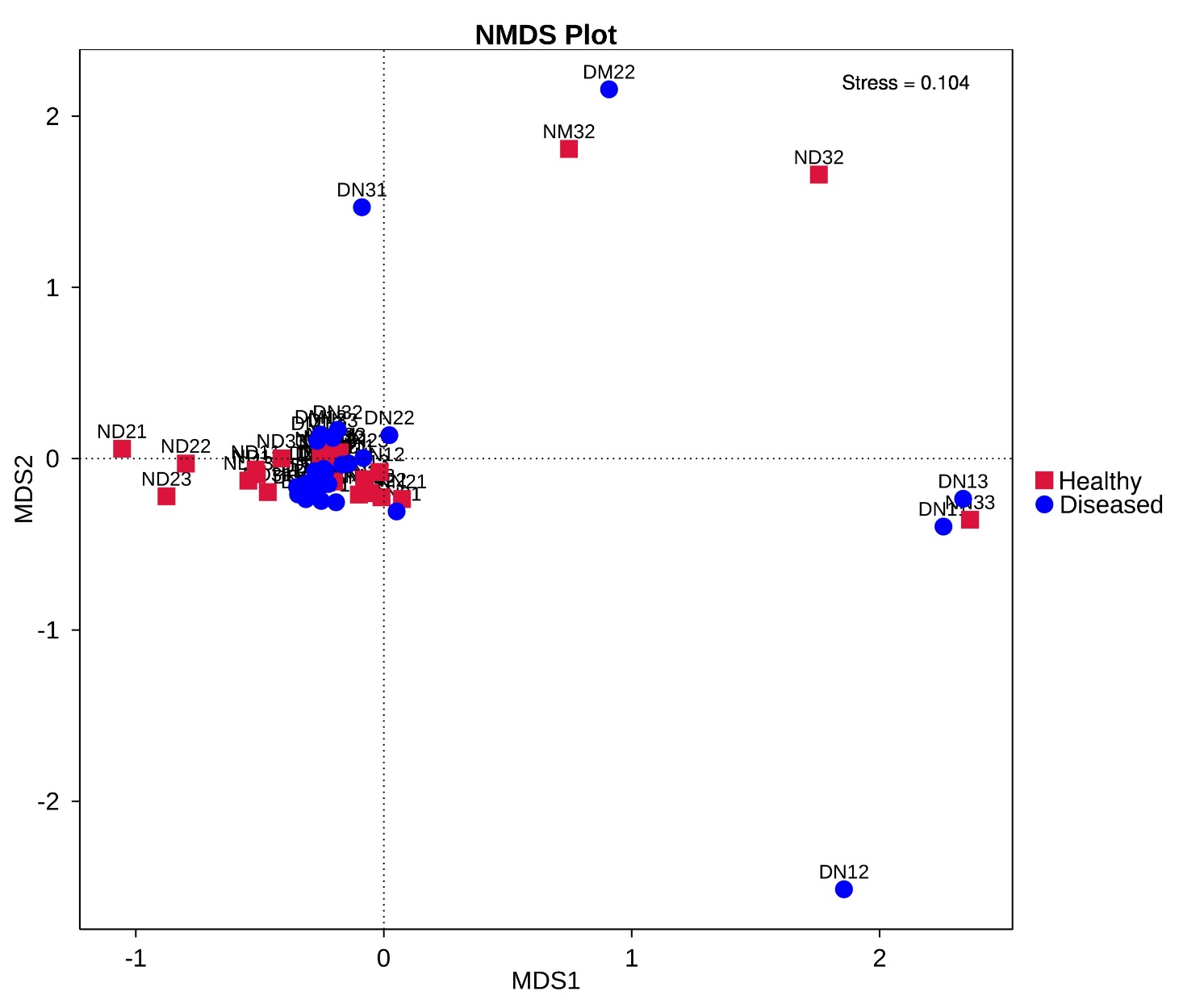


## Supplementary Figure 5. Non-metric multidimensional scaling plots (NMDS) shows the distances of bacterial community between healthy and diseased groups of *H. fossilis*. Five diseased and three healthy samples situated far from the main clusters.


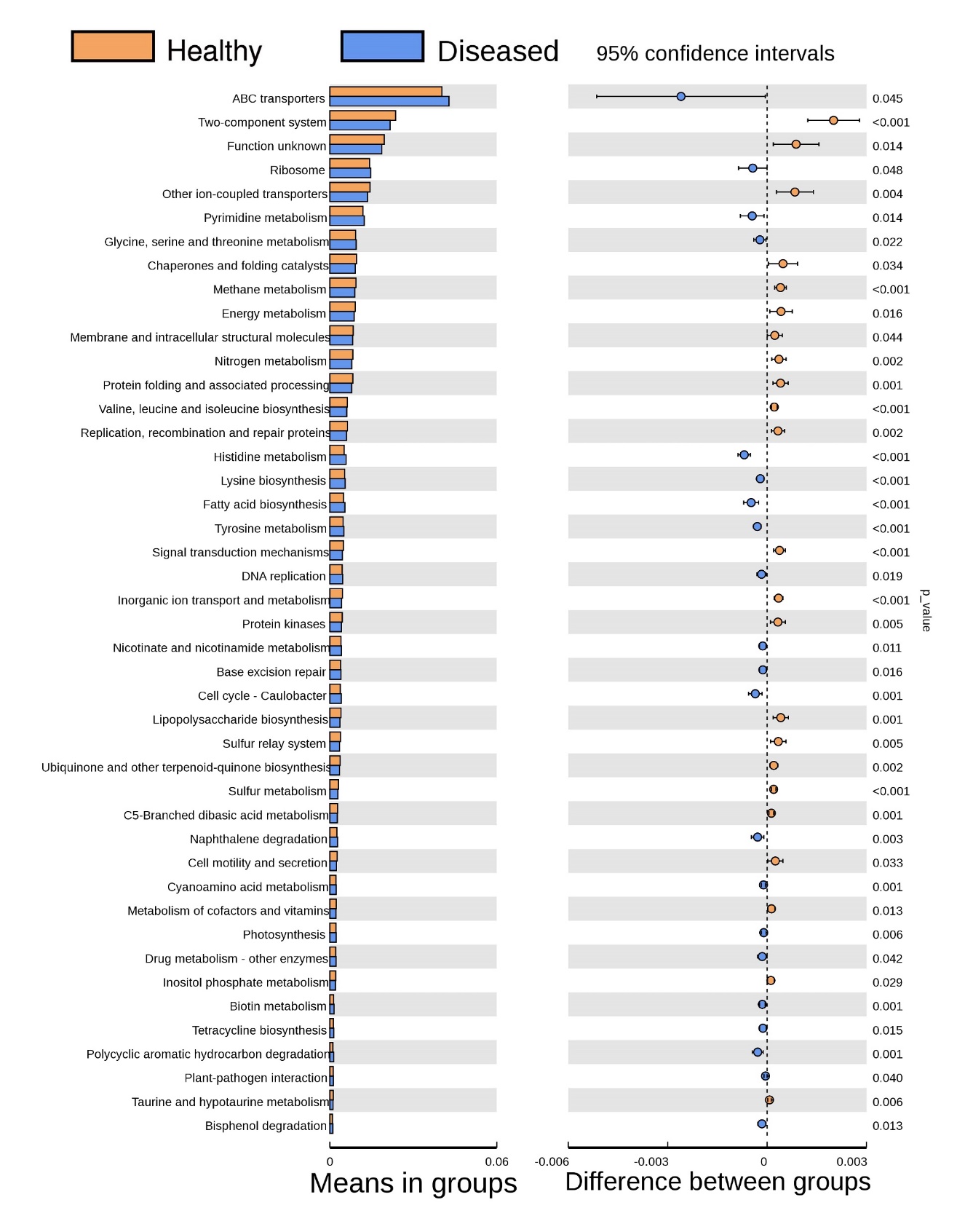


## Supplementary Figure 6. The Welch’s t-test of the functional predictions generated by the KEGG level 3. P values (<0.005) represents the statistically significant variation between the groups.


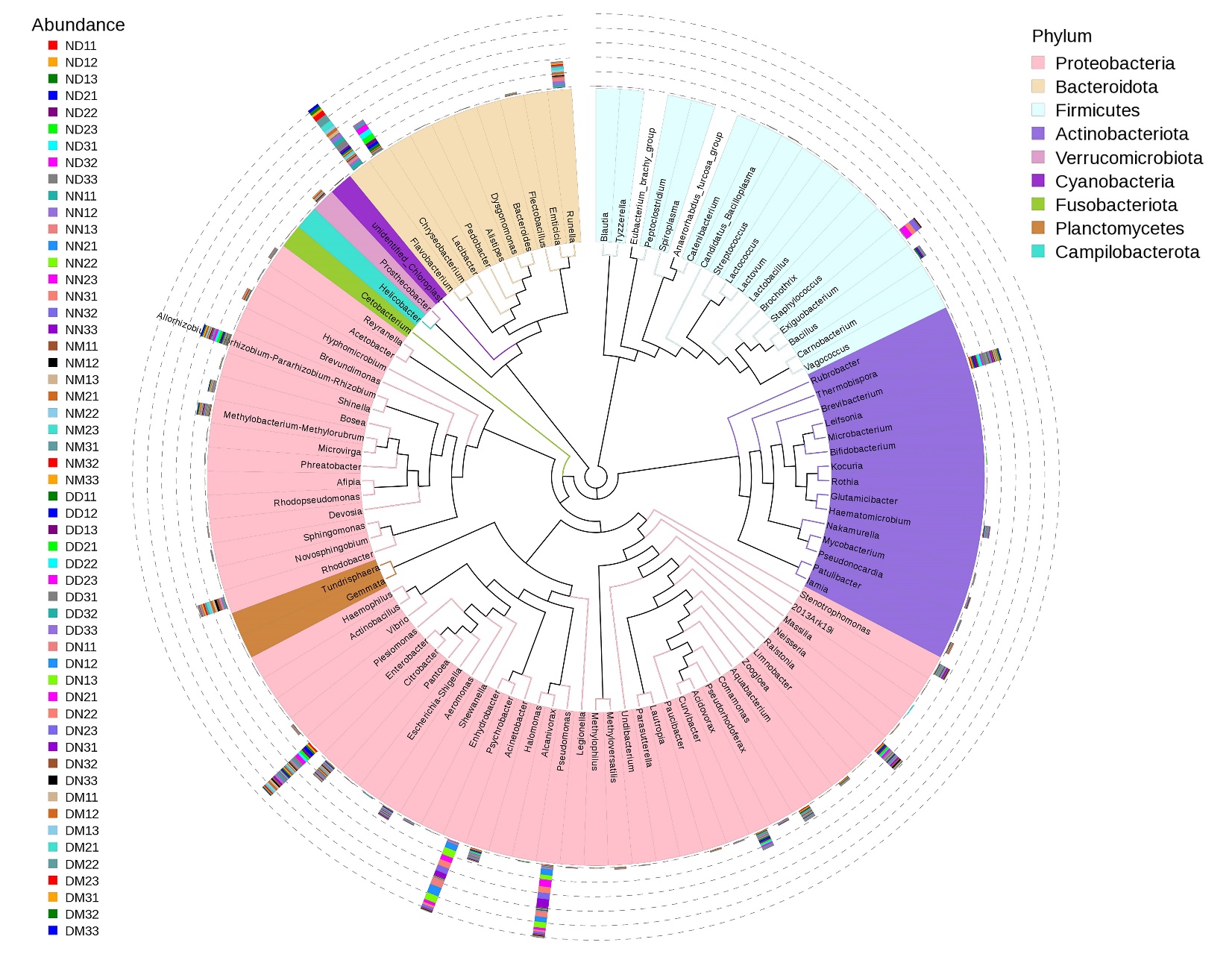


## Supplementary Figure 7. Cladogram shows the abundance of 100 genera in healthy and diseased groups of *H. fossilis*.


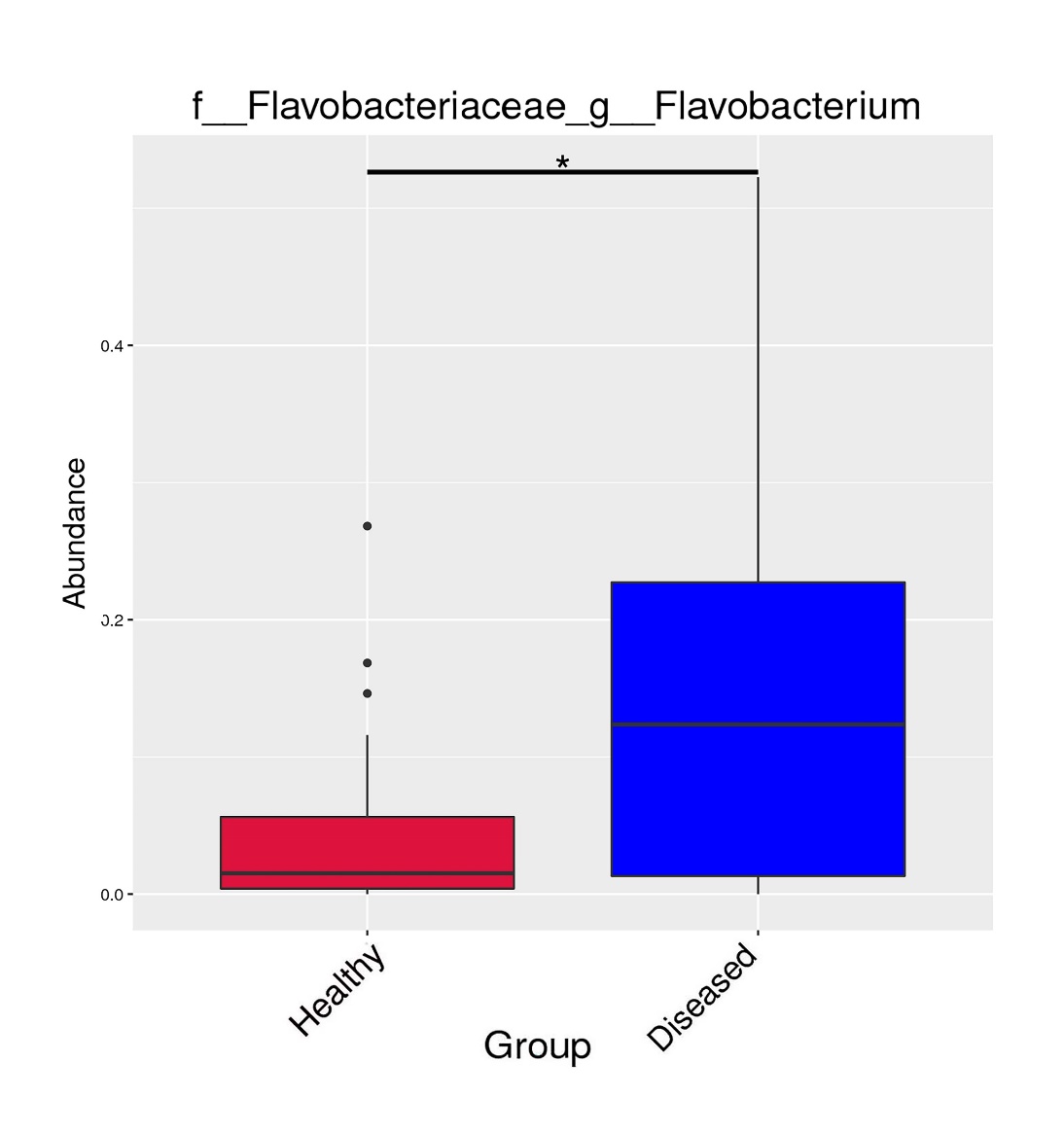


## Supplementary Figure 8. Box plots shows the significant difference of Flavobacterium between healthy and diseased groups of *H. fossilis.*1.2 Supplementary Tables

Supplementary Table 1. Pairwise dissimilarities within the groups by one way analysis of similarities

| Group | R | P |
| --- | --- | --- |
| Dis.Nar-Dis.Mym | 0.7243 | 0.001 |
| Hea.Din-Dis.Mym | 0.9883 | 0.001 |
| Hea.Din-Dis.Nar | 0.608 | 0.002 |
| Hea.Nar-Dis.Mym | 0.7723 | 0.001 |
| Hea.Nar-Dis.Nar | 0.1135 | 0.109 |
| Hea.Nar-Hea.Din | 0.7809 | 0.001 |
| Dis.Din-Dis.Mym | 0.7889 | 0.001 |
| Dis.Din-Dis.Nar | 0.7171 | 0.001 |
| Dis.Din-Hea.Din | 0.9371 | 0.001 |
| Dis.Din-Hea.Nar | 0.7399 | 0.001 |
| Hea.Nar-Dis.Mym | 1.0 | 0.001 |
| Hea.Mym-Dis.Nar | 0.6663 | 0.001 |
| Hea.Mym-Hea.Din | 1.0 | 0.001 |
| Hea.Mym-Hea.Nar | 0.7414 | 0.001 |
| Hea.Mym-Dis.Din | 0.9705 | 0.001 |
